# Seasonal Nutrient Cycling and Enrichment of Nutrient-Related Soil Microbes Aid in the Adaptation of Ramie (*Boehmeria nivea* L.) to Nutrient-Deficient Conditions

**DOI:** 10.3389/fpls.2021.644904

**Published:** 2021-03-31

**Authors:** Shenglan Wu, Shuai Xue, Yasir Iqbal, Hucheng Xing, Yucheng Jie

**Affiliations:** ^1^College of Agronomy, Hunan Agricultural University, Changsha, China; ^2^Orient Science & Technology College of Hunan Agricultural University, Changsha, China; ^3^College of Bioscience & Biotechnology, Hunan Agricultural University, Changsha, China

**Keywords:** fibrous crop, nutrient retranslocation, microbial communities, tolerance strategy, infertile soil

## Abstract

The breeding for varieties tolerant of adverse growing conditions is critical for sustainable agriculture, especially for ramie (*Boehmeria nivea* L.). However, a lack of information on the tolerance of ramie to nutrient-deficient conditions has hindered efforts to breed ramie varieties tolerant of such conditions. The main objective of this study was to explore the tolerance strategies of ramie plants under poor soil conditions using long-term (8–9 years) field trials. Genotypes of Duobeiti 1 and Xiangzhu XB were highly tolerant of poor soil conditions. The contributions of seasonal nutrient cycling and rhizobacteria to the ability of ramie to tolerate poor soil were tested. Nitrogen and phosphorus retranslocation to the root at the end of the growing season helped ramie adapt to poor soil conditions. The contribution of the microbial community was analyzed using high-throughput Illumina MiSeq sequencing technology. The enrichment of beneficial bacteria (mainly *Bradyrhizobium*, *Gaiella*, and *norank_o_Gaiellales*) and the reduction of harmful fungi (mainly *Cladosporium* and *Aspergillus*) also contributed to the ability of ramie to tolerate poor soils. The results of this study provide new insight into the ability of ramie to tolerate adverse conditions and aid future efforts to breed and cultivate ramie tolerant of adverse conditions.

## Introduction

Ramie (*Boehmeria nivea* L.), commonly known as China grass, is a hardy perennial herbaceous plant (Urticaceae: tribe Boehmerieae). Cells in its stem phloem are long, with a maximum length of 60 cm, and are made up of nearly pure cellulose (ca. 96%) (Satya et al., 2019). These long bast cells have traditionally been used as natural fibers to produce a variety of products, such as cloth, finishing net, and upholstery fabrics. With the increase in the demand for natural fibers, ramie has become an important industrial crop; it is mainly cultivated in Asian countries (Rehman et al., 2019). China contributes to more than 96% of the world’s ramie production (FAOSTAT)^1^. However, there has been a considerable decline in the global area under ramie cultivation: from 143,952 ha in 2007 to 52,884 ha in 2018. The decline has been primarily caused by the replacement of ramie with other crops (mainly *Oryza sativa* L.) in China.

The low profitability of ramie production, which stems from the increasing input costs but low and stable fiber price, is the main factor contributing to the reduction in the area under ramie cultivation in China. Multiple (usually two to three) mid-season harvests result in the removal of macroelements (i.e., uptake by the plants) such as nitrogen (N), phosphorus (P), and potassium (K) from the soil (Liu et al., 2013). Consequently, heavy fertilizer application is essential for achieving stable yields and the production of high-quality fiber (Tatar et al., 2010). Ramie cultivation could be improved if fertilizer inputs could be reduced. Although optimizing cultivation techniques (e.g., fertilization or harvesting strategies) could reduce fertilizer input (Liu et al., 2012; Zeng et al., 2013), the breeding for varieties that can use nutrients efficiently and grow on poor soils will provide a more efficient and long-term solution. Additionally, given that ramie shows strong heavy metal tolerance and has a variety of nonfood uses (Rosariastuti et al., 2018; Lan et al., 2020), ramie could be grown in heavy metal-contaminated soil (Neilson and Rajakaruna, 2014; Zheng J. et al., 2019). Heavy metal-contaminated soils (mainly in mining areas) are generally characterized by poor fertility (Mensah, 2015; Lwin et al., 2018). Therefore, expanding ramie cultivation to these areas requires breeding varieties that can tolerate soils with poor fertility.

There has been a little effort to develop new ramie varieties with the potential to grow in poor soil. A lack of information on how ramie plants tolerate nutrient-deficient soil conditions (i.e., their tolerance strategy) has hindered breeding efforts. The aim of this study was to investigate the strategies by which ramie plants cope with poor soil conditions. In perennial plants, nutrient retranslocation (or seasonal nutrient cycling) plays an important role in maintaining their normal growth when nutrients are limited (Fife et al., 2008; Moreira and Fageria, 2009; Erley et al., 2010), especially for plants with extensive underground root systems, such as miscanthus (*Miscanthus* spp.) and giant reed (*Arundo donax* L.) (Nasso et al., 2011, 2013; Ruf et al., 2017). Ramie is a perennial herb characterized by a well-developed underground root system composed of rhizomes, radish roots, and fine roots. One possible tolerance strategy is *via* the seasonal cycling of macronutrients, which could improve the adaptation of ramie to nutrient-deficient conditions. To test for this strategy, relationships between the seasonal rates of change in N, P, and K and ramie’s poor soil tolerance index were characterized. In previous studies, rhizobacteria were found to play a major role in the tolerance of plants to poor soil by mediating the process of N cycling and plant nutrient acquisition (Liu and Ludewig, 2019). The soil microbial community might also contribute to the tolerance of ramie to poor soil. To test for this strategy, the relationship between the structure of the soil microbial community and ramie’s poor soil tolerance index was assessed. The above two strategies were tested using different germplasm materials in two long-term field trials.

## Materials and Methods

### Site Conditions and Experimental Design

This study was conducted by establishing ramie stands at the experimental stations of Changsha (ECS, 28°11′10″N, 113°4′5″E, 58 m a.s.l.) and Huarong (EHR, 29°32′46″N, 112°39′57″E, 73 m a.s.l.). The ECS site is characterized by red clay soil (collected in November 2017) with poor fertility [total nitrogen (TN) 0.69 g/kg, available phosphorus (AP) 9.62 mg/kg, and exchangeable potassium (EP) 56.53 mg/kg], whereas the EHR site is a wasteland with a poor sandy red soil (TN 0.66 g/kg, AP 7.61 mg/kg, and EP 59.54 mg/kg; collected in November 2018). Both sites feature a humid subtropical monsoon climate with a long-term annual average precipitation of 1,420 and 1,062 mm and air temperature of 17.1 and 16.7°C for ECS and EHR, respectively.

Field trials at both sites were conducted in spring 2010. Plant materials used for each trial are shown in Table 1. Experiments were conducted in a randomized block design (block size of 4 × 2.5 m) with three replicates. All materials were planted using 300 g of rhizome (collected from the germplasms garden of our team at Changsha) at a density of four plants/m^2^ (i.e., 0.5 × 0.5 m spacing). Soil tillage and harrowing were conducted before planting. After planting, only two harvests per year (July and December) were carried out. No other management practices (e.g., irrigation, weeding, and pest control) were used.

**TABLE 1:** Information of the ramie materials used in the two field trials.

Material name	Abbreviation	Material type	Used in trial
Xiangzhu X1	XZ-X1	Genotype	ECS trial
Xiangzhu X2	XZ-X2	Genotype	ECS trial and EHR trial
Xiangzhu X3	XZ-X3	Genotype	ECS trial
Xiangzhu XB	XZ-XB	Genotype	ECS trial and EHR trial
Xiangzhu 3	XZ-3	Variety	ECS trial and EHR trial
Duobeiti 1	D-1	Genotype	ECS trial
Zhongzhu 1	ZZ-1	Variety	EHR trial

*ECS and EHR represent the trials established at the experimental stations of Changsha and Huarong, respectively.*

### Sampling Strategy and Data Collection

#### Evaluation of Ramie Field Performance

For the ECS trial, the field performance of established plants was measured in November of both the 2010 (the establishment year) and 2018 growing seasons. Field performance was also assessed in the middle of July 2018 near the mid-season harvest. Similarly, morphological features of the EHR plants were measured in mid-July and the end of November 2019. The morphological characteristics included plant height, tiller number, and aboveground and belowground biomass. The heights of 10 randomized selected plants within each plot were recorded. At each sampling event, an area of 0.5 m^2^ in each plot was randomly chosen using a 0.5 × 1-m quadrat so that the distance between the quadrat boundary and plot boundary was no less than 30 cm. The aboveground biomass within the quadrat was then harvested. In addition, the belowground root system, including the rhizome, radish root, and fine root (hereafter referred to collectively as “root”) was excavated (to a depth of 40 cm), washed, and collected. All of the biomass collected was dried to a constant weight of 60°C and then weighed to collect data on biomass yield.

#### Evaluation of the Plant Nutrient Concentration

For the ECS field trial, plant nutrient concentration was analyzed based on samples that were collected monthly from April to December 2018. In the middle of each sampling month, aboveground and belowground biomass within 0.5 m^2^ was harvested as described above. The aboveground plants were separated into stem and leaves. All subsamples were dried to a constant weight at 60°C. Afterward, each dried sample was weighed and milled to powder (100 mesh) for subsequent nutrient concentration analysis. The nitrogen concentration was determined by the Kjeldahl method using a K9840 distillator/titrator (Haineng, China). The phosphorus concentration was determined with a UV-2700i spectrophotometer (Shimadzu, Japan) at 700 nm using the molybdenum blue method. The K concentrations were analyzed with the flame photometry method using an FP6450 photometer (SHjingmi, China). These analysis methods are described in detail in the work of Bao (2000). Samples for all nutrient analyses were subjected to H_2_SO_4_-H_2_O_2_ digestion. Each sample was analyzed three times, and the mean value was used in the analyses.

#### Soil Microbial Composition Analysis

In this study, soil microbial composition analysis was conducted using the soil collected from the EHR trial in October 2019. High-throughput Illumina MiSeq sequencing technology was used to analyze the structure of soil microbial (including fungi and bacteria) communities. Five plants from each plot were randomly selected according to the “S” shape principle. Their rhizosphere soils were then collected to prepare a composite sample by removing and mixing the soil from rhizomes and roots. Composite soil was mixed, sieved (2 mm), reduced to 50 g, and finally stored in liquid nitrogen until high-throughput sequencing analysis.

High-throughput sequencing analysis generally involves three steps: DNA extraction, PCR amplification, and Illumina MiSeq sequencing. DNA of the total microbial community was first isolated from the aforementioned samples using the E.Z.N.A.^®^ soil DNA Kit (Omega Bio-tek, Norcross, GA, United States) as per the manufacturer’s instructions. The DNA extract was then tested on 1% agarose gel, and DNA concentration and purity were determined with a NanoDrop 2000 UV-Vis spectrophotometer (Thermo Scientific, Wilmington, MA, United States). The tested DNA samples were amplified using an ABI GeneAmp^®^ 9700 PCR thermocycler (ABI, CA, United States). The primer pairs of 338F/806R were used to amplify the hypervariable region V3-V4 of the bacterial 16S rRNA gene and ITS1F/ITS2R to the internal transcribed spacer (ITS) region of fungal rRNA genes. Both PCR reactions were performed as follows: initial denaturation at 95°C for 3 min; 27 cycles of denaturation at 95°C for 30 s, annealing at 55°C for 30 s, and extension at 72°C for 45 s; and a final extension at 72°C for 10 min. The TransGen AP221-02 (TransStart Fastpfu DNA Polymerase) mixture and TaKaRa rTaq DNA Polymerase mixture were used for the PCR of the bacterial 16S rRNA gene and fungal rRNA genes, respectively; details of the procedure are provided in Zhang et al. (2019). The PCR products were extracted from a 2% agarose gel and purified using the AxyPrep DNA Gel Extraction Kit (Axygen Biosciences, Union City, CA, United States) as per the manufacturer’s instructions and quantified using a Quantus^TM^ Fluorometer (Promega, United States). Purified amplicons were pooled in equimolar amounts and paired-end sequenced (2 × 300) on an Illumina MiSeq platform (Illumina, San Diego, United States) as per the standard protocols of Majorbio Bio-Pharm Technology Co. Ltd. (Shanghai, China).

### Data Analysis

#### Comparison of Ramie Field Performance and Nutrient Concentration Between Genotypes

Field performance and nutrient concentration among genotypes were compared using ANOVA (analysis of variance) in SAS 9.4 software (SAS Institute, Cary, NC, United States). Different plant genotypes and data collection times were considered independent variables. The mean of each trait was tested at the *p* < 0.05 level using Duncan’s multiple range test.

Evaluation of the overall field performance is a multi-criteria decision-making process that involves many factors. In this study, a membership function-derived normalized field performance index (NFPI) was used to comprehensively express overall field performance (Kang et al., 2012). The NFPI value of each field performance trait was calculated based on the following formula:

$${\text{y}}_{i}\,\left(k\right)=\frac{x_{i}\,\left(k\right)-\min x\,\left(k\right)}{\max x\,\left(k\right)-\min x\,\left(k\right)}$$

where *y*_*i*_(*k*) represents the NFPI value of the *i*th genotype in terms of the *k*th field performance trait, *x*_*i*_(*k*) denotes the field-recorded value of the *i*th genotype in terms of the *k*th field performance trait, and max *x*(*k*) and min *x*(*k*) represent the largest and smallest value of *x*_*i*_(*k*), respectively.

The overall NFPI of individual germplasm represents the average value of all calculated *y*_*i*_(*k*) values, as field performance indicators were assumed to make an equal contribution to the overall NFPI.

#### Analysis of the Nutrient Changes Affecting the Poor Soil Tolerance Ability of Ramie

Correlation analysis (hereafter referred to as CA analysis) was used to evaluate how nutrient cycling contributes to the poor soil tolerance ability of ramie. The poor soil tolerance ability of each germplasm was defined as its mean overall NFPI in the two 2018 growth periods. The independent variables of the CA analysis were the nutrient change indicators, and the dependent variable was the overall NFPI of the tested genotypes. The nutrient change indicators included nine from the first growth period (GP1: April–July) and nine from the second growth period (GP2: August to December). The first nine were the rates of change in the concentration of the three tested nutrients (N, P, and K) in the three plant parts (leaves, stem, and root) over GP1 (i.e., July relative to April). The other nine were the rates of change in the concentration of these nutrients over GP2 (i.e., December relative to August). To reduce the dimensions of the complex indicators, principal component analysis (PCA) was carried out prior to the CA analysis to identify the most important variables relevant to nutrient change. Only indicators with significant (*p* < 0.05) correlations with principal components were used in the CA analysis. Based on the PCA results, 10 variables were selected, which were the rates of change in the concentration of N, P, and K in the root in GP2 (hereafter referred to as RN2, RP2, and RK2, respectively); rates of change in the concentration of N and P in the stem in GP1 (SN1 and SP1, respectively); rates of change in the concentration of N and K in the stem in GP2 (SN2 and SK2, respectively); rates of change in the concentration of P and K in the leaves in GP1 (LP1 and LK1, respectively); and the rate of change in the concentration of K in the root in GP1 (RK1). Correlations between the ramie’s overall NFPI and the above 10 indicators were performed using the CORR procedure in SAS 9.4 software. Pearson’s correlation coefficients and their significance were used to assess the strength of the correlations between NFPI and the 10 indicators.

#### Microbial Composition Analysis

The raw FASTQ sequencing reads were first demultiplexed, quality-filtered by Trimmomatic, and merged by FLASH following the criteria of Zhang et al. (2019). The structure of the soil microbial community was then analyzed in the free online Majorbio Platform^2^, which is based on the R packages USEARCH (Version 7.0), PICRUSt (Version 1.1.0), UNITE (Version 8.0), and FUNGuild (Version 1.0).

## Results

### Evaluation of the Poor Soil Tolerance of Different Ramie Genotypes Based on Their Field Performance

The long-term plant field performance under poor soil conditions was evaluated assuming that a higher field performance corresponded to a stronger ability to tolerate poor soil conditions. In the ECS trial, the genotype (G), plantation year (Y), and growth period (GP) had a significant effect on all of the morphological traits measured (Table 2). Plants in November 2018 (eighth plantation year) were on average 23.1% lower in height (69.5 vs. 90.4 cm, *p* < 0.001 for the effect of Y), had 4.1 times more tillers (71.5 vs. 14, *p* < 0.001), and had 53.1% higher aboveground yield (179.0 vs. 116.9 g/m^2^, *p* < 0.001) compared with plants in November 2010 (the establishment year). Differences in the tested traits (Table 2) between the first and second growth periods were also significant (*p* < 0.01); specifically, plants at the end of GP1 were generally superior in terms of morphology compared with GP2 plants, with the exception of belowground yield. The belowground yield of the six genotypes was 2.08 kg/m^2^ on average at the end of GP2, which was 35.9% higher than that (1.53 kg/m^2^) at the end of GP1. There were significant differences (*p* < 0.001 for the effect of G) in all morphological traits across the six tested genotypes. However, the ability to tolerate poor soil conditions (i.e., the overall NFPI) was not consistent over the years and also differed between growth periods (Table 3). At the end of 2010, XZ-3 had the highest overall NFPI (0.786), whereas XZ-X2 had the lowest overall NFPI (0.016). The other four genotypes showed a similar performance. However, in 2018, D-1 had the highest overall NFPI (0.667) at the end of GP1, followed by XZ-X2 (0.406); at the end of GP2, D-1 had a lower NFPI (0.716) than XZ-XB (0.799). A synthetic NFPI was used to comprehensively evaluate field performance, which was defined as the average value of the overall NFPI in GP1 and GP2. The genotypes from high performance (i.e., a better ability to thrive under poor soil conditions) to low performance were D-1, XZ-XB, XZ-3, XZ-X2, XZ-X3, and XZ-X1.

**TABLE 2:** Comparison of the field performance of the six tested ramie genotypes in the field trial at Changsha (ECS trial).

Material	2010 November			2018 July (end of 2018 GP1)				2018 November (end of 2018 GP2)			
	Plant height (cm)	Tiller number/m^2^	Aboveground yield (g/m^2^)	Plant height (cm)	Tiller number/m^2^	Aboveground yield (g/m^2^)	Belowground yield (kg/m^2^)	Plant height (cm)	Tiller number/m^2^	Aboveground yield (g/m^2^)	Belowground yield (kg/m^2^)
XZ-3	99.3 B	16.0 G	136.0 C	145.1 b	94.3 bc	378.9 c	1.60 d	76.1 DEef	66.3 De	214.4 Ae	1.69 cd
D-1	115.7 A	12.0 G	100.4 D	178.4 a	48.0 g	687.5 a	1.40 d	87.1 Ce	42.7 Fh	203.5 Aef	1.91 bc
XZ-X1	77.3 DE	16.0 G	134.1 C	124.2 d	96.7 b	398.9 bc	1.43 d	54.4 Fg	108.0 Aa	125.0 Ch	1.56 d
XZ-X2	78.5 D	16.0 G	91.9 E	128.1 c	91.7 c	382.4 c	1.82 c	64.4 Eg	55.0 Eg	174.8 Bg	2.29 b
XZ-X3	79.3 CD	12.0 G	121.5 C	122.4 d	84.3 cd	404.8 b	1.45 d	56.9 Fg	83.0 Bd	170.8 Bg	2.21 bc
XZ-XB	92.3 BC	12.0 G	117.6 CD	142.3 b	64.0 ef	342.1 d	1.50 d	78.2 Def	73.7 Cde	185.7 Bf	2.84 a
**Sources of variance in ANOVA (*p* > *F*)**											
G	<0.001	<0.001	<0.001				G	<0.001	<0.001	<0.001	0.0014
Y	<0.001	<0.001	<0.001				GP	<0.001	0.0006	<0.001	<0.001
G × GP	0.5211	<0.001	<0.001				G × GP	0.1047	<0.001	<0.001	0.0056

*Abbreviations of plant materials: XZ-3, Xiangzhu 3; D-1, Duobeiti 1; XZ-X1, Xiangzhu X1; XZ-X2, Xiangzhu X2; XZ-X3, Xiangzhu X3; XZ-XB, Xiangzhu XB. Abbreviations in ANOVA source: G, ramie genotype; Y, data collection year; GP, ramie growth period within 1 year with GP1 (from April to July) and GP2 (from August to December) (i.e., plant harvested at the end of July). Different capital letters within each measured trait indicate significant differences at the *p* < 0.05 level between the trait measured in 2010 November and 2018 November. Different small letters within each measured trait indicate significant differences at the *p* < 0.05 level between the trait measured in 2018 July and 2018 November.*

**TABLE 3:** The normalized field performance index (NFPI) and order of the six ramie germplasms tested in the field trial at Changsha (ECS trial).

Material	2010 November		2018 July (end of 2018 GP1)		2018 November (end of 2018 GP2)		2018 average	
	NFPI	Order	NFPI	Order	NFPI	Order	NFPI	Order
XZ-3	0.786	1	0.331	3	0.589	3	0.460	3
D-1	0.596	2	0.667	1	0.716	2	0.691	1
XZ-X1	0.478	4	0.091	6	0.000	6	0.045	6
XZ-X2	0.016	6	0.406	2	0.477	4	0.441	4
XZ-X3	0.362	5	0.101	5	0.366	5	0.233	5
XZ-XB	0.487	3	0.201	4	0.799	1	0.500	2

*Abbreviations of plant materials: XZ-3, Xiangzhu 3; D-1, Duobeiti 1; XZ-X1, Xiangzhu X1; XZ-X2, Xiangzhu X2; XZ-X3, Xiangzhu X3; XZ-XB, Xiangzhu XB. NFPI of 2010 November was calculated based on the plant height and aboveground yield measured in 2010 November. NFPI of 2018 July and 2018 November was calculated based on the plant height, aboveground yield, and belowground yield measured in 2018 July and November, respectively. NFPI of 2018 average is the mean of NFPI in 2018 July and 2018 November.*

Although genotype and growth period still had a significant (*p* < 0.001) effect on ramie’s field performance in the EHR trail, their effects were not fully consistent with those observed in the ECS trial (Table 4). The plants harvested at the end of GP2 were significantly (*p* < 0.001) taller than the plants at the end of GP1 (182.0 vs. 148.1 cm) but had fewer tillers (28.5 vs. 37.8, *p* = 0.003), which is in contrast to the effect of growth period in the ECS trial. Additionally, the aboveground yield did not significantly differ between the two growth periods (*p* = 0.458). The comprehensive performance evaluation revealed that different genotypes performed similarly across the two growth periods, and the genotypes from high performance to low performance were XZ-XB, ZZ-1, XZ-X2, and XZ-3. This pattern is generally consistent with that in the ECS trial, in which XZ-XB was the best-performing genotype (synthetic NFPI = 0.997), and XZ-3 was the worst-performing genotype (synthetic NFPI = 0.000).

**TABLE 4:** Comparison of the field performance of the four tested ramie genotypes in the field trial at Huarong (EHR trial).

Material	2019 July (end of 2019 GP1)				2019 November (end of 2019 GP2)				2019 average	
			
	Plant height (cm)	Tiller number/m^2^	Aboveground yield (g/m^2^)	NFPI	Plant height (cm)	Tiller number/m^2^	Aboveground yield (g/m^2^)	NFPI	NFPI	Order
XZ-3	121.7 e	28.3 cd	297.9 c	0.000	159.1 cd	20.0 d	219.6 d	0.000	0.000	4
ZZ-1	156.1 cd	44.7 a	365.7 b	0.685	181.3 b	34.7 b	430.2 a	0.701	0.693	2
XZ-X2	146.3 d	43.3 a	386.9 ab	0.680	173.4 bc	32.0 c	355.5 b	0.452	0.566	3
XZ-XB	168.4 c	35.0 b	404.8 ab	1.000	214.3 a	27.3 cd	410.6 ab	0.953	0.977	1
**Sources of variance in ANOVA (*p* > *F*)**										
G	<0.001	0.0002	<0.001							
GP	<0.001	0.0003	0.4580							
G × GP	0.3510	0.9186	0.0094							

*Abbreviations of plant materials: XZ-3, Xiangzhu 3; ZZ-1, Zhongzhu 1; XZ-X2, Xiangzhu X2; XZ-XB, Xiangzhu XB. NFPI (normalized field performance index) of 2019 July and 2019 November was calculated based on the plant height and aboveground yield measured in 2019 July and November, respectively. NFPI of 2019 average is the mean of NFPI in 2019 July and 2019 November. Abbreviations in ANOVA source: G, ramie genotype; GP, ramie growth period within 1 year with GP1 (from April to July) and GP2 (from August to December) (i.e., plant harvested at the end of July). Different small letters within each measured trait indicate significant differences at the *p* < 0.05 level.*

### Relationship Between Seasonal Nutrient Cycling and Ramie Field Performance

**FIGURE 1:**
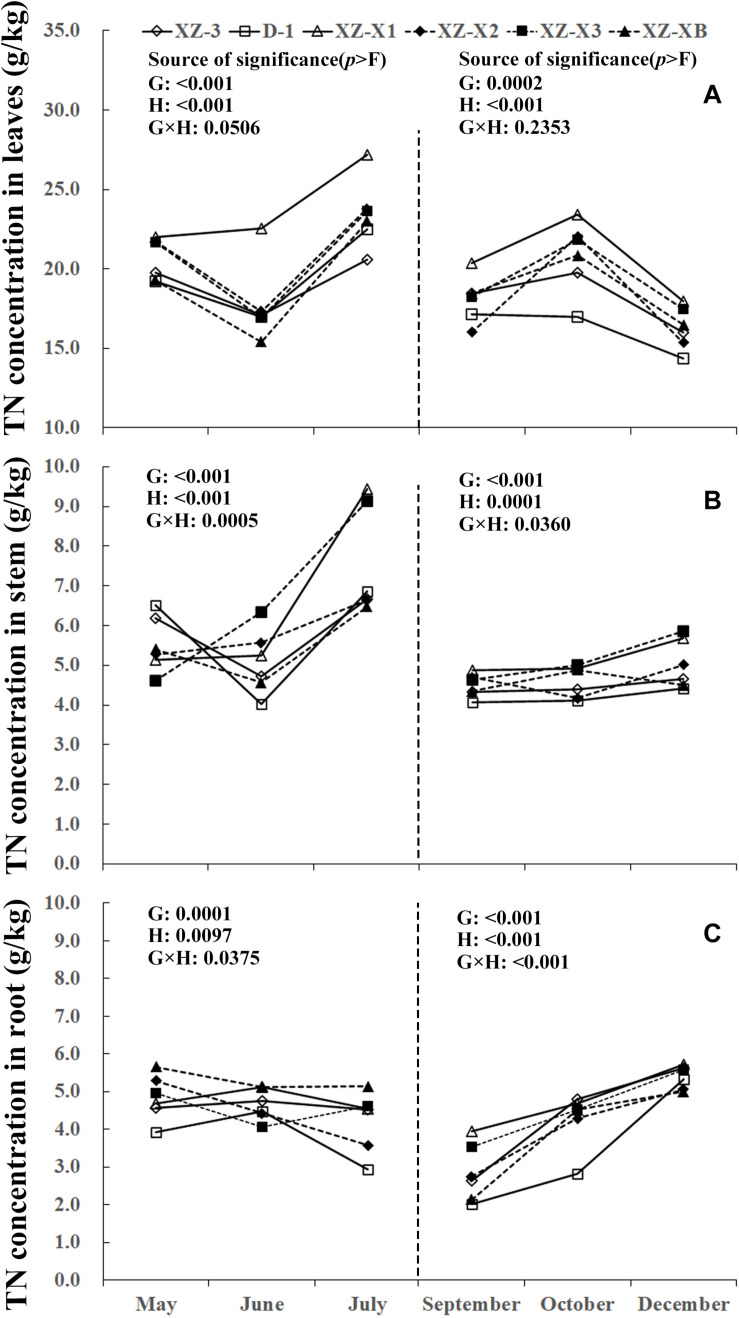
Seasonal dynamics of total nitrogen (TN) concentration in the leaves **(A)**, stem **(B)**, and root **(C)** of different ramie genotypes tested in the ECS trial. Genotype abbreviations of XZ-3, D-1, XZ-X1, XZ-X2, XZ-X3, and XZ-XB correspond to Xiangzhu 3, Duobeiti 1, Xiangzhu X1, Xiangzhu X2, Xiangzhu X3, and Xiangzhu XB, respectively. G and H represent genotype and data collection time, respectively.

**FIGURE 2:**
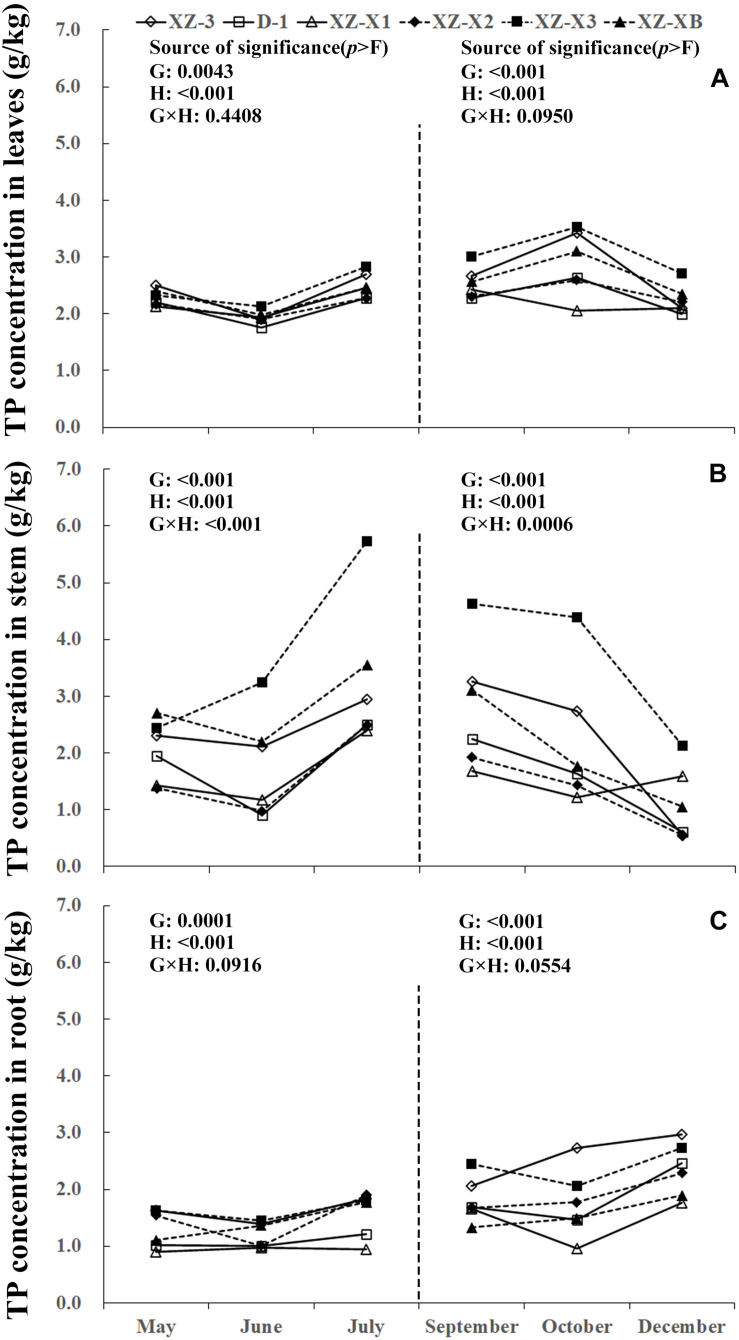
Seasonal dynamics of total phosphorus (TP) concentration in the leaves **(A)**, stem **(B)**, and root **(C)** of different ramie genotypes tested in the ECS trial. Genotype abbreviations of XZ-3, D-1, XZ-X1, XZ-X2, XZ-X3, and XZ-XB correspond to Xiangzhu 3, Duobeiti 1, Xiangzhu X1, Xiangzhu X2, Xiangzhu X3, and Xiangzhu XB, respectively. G and H represent genotype and data collection time, respectively.

**FIGURE 3:**
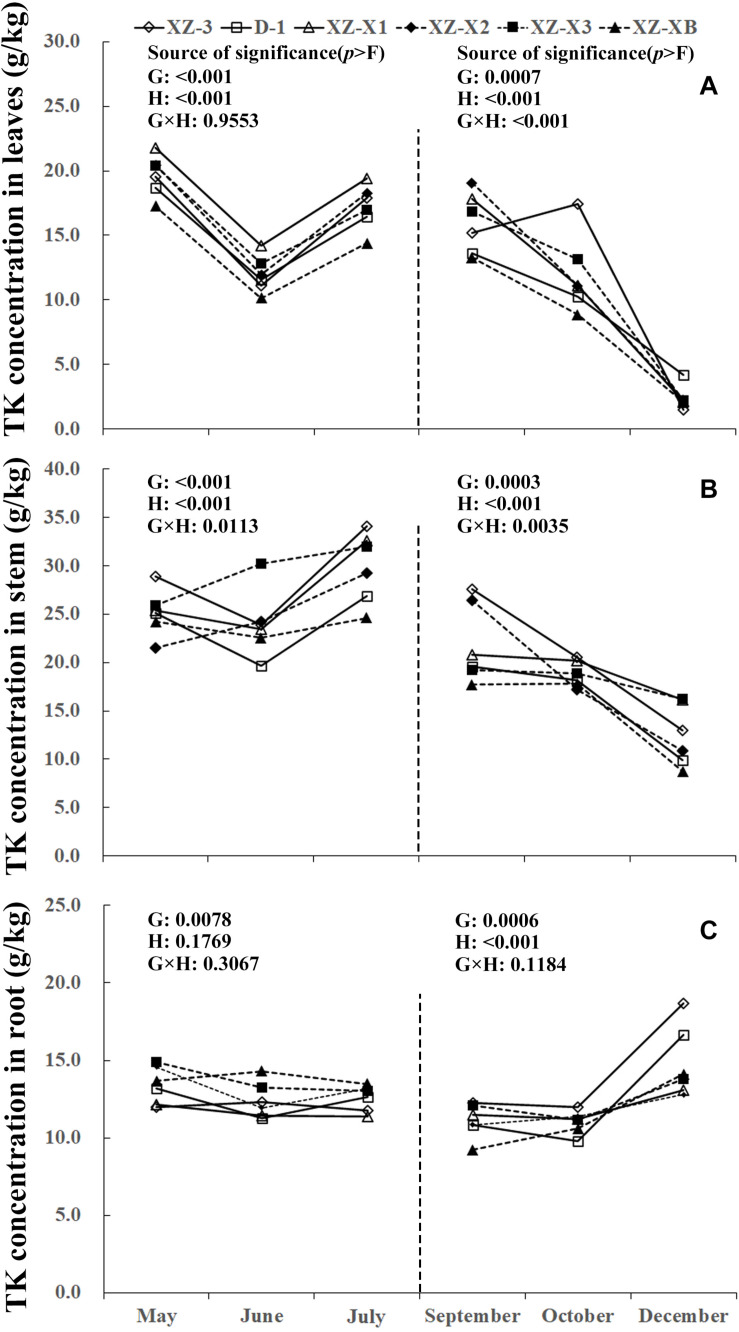
Seasonal dynamics of total potassium (TK) concentration in the leaves **(A)**, stem **(B)**, and root **(C)** of different ramie genotypes tested in the ECS trial. Genotype abbreviations of XZ-3, D-1, XZ-X1, XZ-X2, XZ-X3, and XZ-XB represent Xiangzhu 3, Duobeiti 1, Xiangzhu X1, Xiangzhu X2, Xiangzhu X3, and Xiangzhu XB, respectively. G and H represent genotype and data collection time, respectively.

A similar pattern of change throughout the growing season was observed for TN, total phosphorus (TP), and total potassium (TK) (Figures 1–3). The nutrient concentration in the leaves and stems increased over time during GP1 (from April to July) but decreased during GP2 (from August to December). For example, at the end of GP1 (July), the average TN concentration was 19.2% higher in the leaves (Figure 1A) (23.0 g/kg) compared with the beginning of May (19.3 g/kg) but significantly decreased from 18.1 g/kg at the beginning of GP2 (September) to 16.2 g/kg at the end of GP2 (December) (*p* < 0.001). Nutrient concentration in the root was relatively stable during GP1 but significantly increased over time in GP2 (Figures 1C, 2C, 3C). From May to July, the TK concentration in the root did not differ significantly (*p* = 0.1769). The concentration of all three tested macronutrients in the root significantly increased from September to December by 89.8% (TN), 29.8% (TP), and 33.6% (TK).

Patterns of nutrient dynamics were complex and varied between genotypes (Figures 1–3). However, there was a general pattern in which genotypes that had a stronger tolerance of poor soil showed gradual increases in the P concentration of the leaves and N concentration of the stems during GP1 but rapid increases in the N, P, and K concentration of the root during GP2. In July 2018, the poorly performing genotype XZ-X3 had significantly (*p* = 0.0043) higher TP concentration in the leaves (2.82 g/kg) compared with the other genotypes, as well as the highest LP1 (21.0%). The best-performing genotype D-1 had a low LP1 (4.0%). Similar changes were also observed for TN in the stem, as the lowest SN1 (5%) was recorded for D-1, followed by XZ-3 (8.0%) and XZ-XB (20.0%); the worst-performing genotype XZ-X1 had a high SN1 (84.0%). During GP2, the highest RN2 (163.0%) was recorded for D-1, whereas the lowest RN2 (45.0%) was recorded for XZ-X1. The highest RP2 (46.0%) and RK2 (54.0%) in the root were also recorded for D-1, whereas the lowest RP2 (6.0%) was recorded for XZ-X1, and the lowest RK2 (14.0%) was recorded for XZ-X3.

Nutrient concentration dynamics and synthetic NFPI in 2018 were analyzed by Pearson’s correlation to determine the contribution of nutrient dynamics to improving the tolerance of ramie to poor soil conditions. The correlation results are shown in Table 5. Synthetic NFPI was significantly positively correlated with RN2 (*r* = 0.946, *p* = 0.004) and RP2 (*r* = 0.932, *p* = 0.007) but significantly negatively correlated with SN1 (*r* = −0.877, *p* = 0.022). The positive correlation between RK2 and synthetic NFPI was marginally significant (*r* = 0.795, *p* = 0.059). The negative correlations of LP1 and SK2 with synthetic NFPI were also marginally significant (*r* = −0.791, *p* = 0.061; *r* = −0.773, *p* = 0.071, respectively).

**TABLE 5:** Correlations between the nutrient change indicator and the ability of ramie to tolerate poor soil.

	Nutrient change indicators									
	RN2	SN1	SN2	RP2	SP1	LP1	RK1	RK2	SK2	LK1
*r*	0.946	−0.877	−0.679	0.932	−0.597	−0.791	0.455	0.795	−0.773	0.042
*p*	0.004	0.022	0.138	0.007	0.211	0.061	0.364	0.059	0.071	0.934

**r*, Pearson’s coefficients; *p*, *p-*values of the Pearson’s coefficients. The first letters of the nutrient change indicators R, S, and L represent the nutrient concentration in the root, stem, and leaves, respectively. The middle letters N, P, and K correspond to nitrogen, phosphorus, and potassium, respectively. The last two numbers (1 or 2) represent the nutrient change in either the first growth period or the second growth period, which is defined as from April to July and from August to December, respectively.*

### Relationship Between Soil Microbe Structure and Ramie Field Performance

**FIGURE 4:**
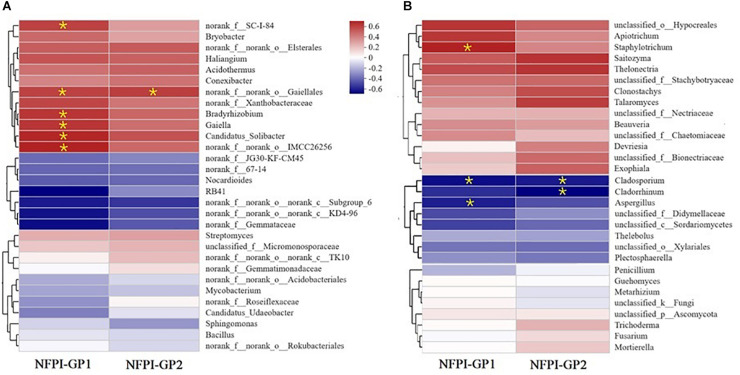
Correlations between the rhizosphere soil microbial community structure and the tolerance of ramie to poor soil conditions (expressed by the normalized field performance index NFPI) during GP1 (from April to July 2019) and GP2 (from August to December 2019) at Huarong experimental station (i.e., EHR trial). The structure of soil microbial communities is expressed at the genus level of bacteria **(A)** and fungi **(B)**. Significantly correlated (*p* < 0.05) relationships are marked by *.

The contribution of soil microbial communities to the ability of ramie to tolerate poor soil was expressed by the relationship between soil microbial community structure (at the genus level) and the ramie field performance index NFPI (during GP1 and GP2) at the EHR site. Only the top 30 genera in terms of abundance for bacterial and fungal communities were considered (Figures 4A,B, respectively). All 30 genera of both bacterial and fungal communities were clustered into three groups: genera showing positive correlations with NFPI, genera showing negative correlations with NFPI, and genera unrelated to NFPI. There were six bacterial genera that were significantly positively correlated with ramie NFPI during GP1 (Pearson correlation coefficients in parentheses, *p* < 0.05): *IMCC26256* (Acidimicrobiia) (0.656), *Candidatus_Solibacter* (0.642), *Bradyrhizobium* (0.604), *Gaiella* (0.603), *norank_f_SC-I-84* (0.589), and *norank_o_Gaiellales* (0.581). During GP2, only *norank_o_Gaiellales* was significantly positively correlated with ramie field performance (*p* < 0.05, *r* = 0.582). Seven genera were negatively correlated with ramie field performance, but none of these correlations were significant (Figure 4A).

Four fungal genera were significantly correlated with ramie field performance, including three negatively correlated genera and one positively correlated genus (Figure 4B). *Cladosporium* was the fungal genus that was the most strongly negatively correlated with NFPI during both GP1 (*r* = −0.659, *p* < 0.05) and GP2 (*r* = −0.628, *p* < 0.05). This genus was highly abundant (20.3%) in the rhizosphere soil of the worst-performing genotype (XZ-3); by contrast, its abundance was only 1.33% in the rhizosphere soil of the best-performing genotype (XZ-XB). The genus *Cladorrhinum* had the lowest correlation coefficient (*r* = −0.72, *p* < 0.05) with NFPI, but this was only for GP2. This is also similar to *Aspergillus*, which only showed a significant negative correlation with NFPI during GP1 (*r* = −0.623, *p* < 0.05). Only *Staphylotrichum* had a significant positive correlation with NFPI during GP1 (*r* = 0.628, *p* < 0.05). Additionally, *Beauveria* was dominant in the soil of the best-performing genotype XZ-XB (25.96%) but was not detected in the soil of the other genotypes.

## Discussion

### Optimal Timing for Evaluating Ramie Field Performance

The first aim of this study was to evaluate the ability of different ramie genotypes to tolerate poor soil based on their field performance. The results of the ECS trial revealed that differences in plant field performance among genotypes between the establishment year and the eighth year after planting were not consistent. The fact that the perennial plants were not fully developed when they were established might explain these differences. Perennial plants generally need a few years to reach a stable level of performance (Jones et al., 2016; Xu et al., 2017; Stolarski et al., 2018). For example, comparison of morphological traits between different *Miscanthus* (a rhizomatous perennial species) genotypes has been generally conducted using data collected after the third year of planting (Xiang et al., 2018; Zheng C. et al., 2019). This should also be the case for perennial ramie. Angelini and Tavarini (2013) showed that stable field performance of ramie is generally achieved between the third to the 13th year after planting. This indicates that morphological traits should be measured from the third to the 13th year after cultivation when comparing differences in the field performance of different ramie genotypes. The results of both trials revealed significant performance differences between harvest times. In the ECS trial, plants performed better at the end of GP1 than at the end of GP2, which is consistent with the results of a previous study (Angelini and Tavarini, 2013). However, the opposite pattern was observed in the EHR trial. These results indicate that the plant phenotype throughout the entire growth period (divided by the mid-season harvest) within a year should be considered to comprehensively evaluate ramie performance. However, if more than two mid-season harvests are conducted, the contribution of each growth period should be weighed separately without taking the average given that ramie generally shows higher performance in early harvests compared with later harvests, especially under three to four mid-season harvest treatments (Liu et al., 2013; Wang et al., 2018).

### Effect of Seasonal Nutrient Cycling on the Ability of Ramie to Thrive Under Nutrient-Deficient Conditions

As expected, N, P, and K retranslocation to the root at the end of the growing season helped ramie plants adapt to poor soil conditions. Our results also support the idea that nutrient retranslocation to the root is a survival strategy for perennial rhizomatous plants to adapt to nutrient-deficient conditions (Fife et al., 2008; Moreira and Fageria, 2009; Erley et al., 2010). However, delayed harvest is necessary for this strategy to be effective given that it provides sufficient time for the plant to complete the retranslocation process before harvest. However, this harvest regime is only recommended for cultivating ramie for ecological purposes, such as phytoremediation. This is mainly because delayed harvest damages ramie’s fiber quality (Liu et al., 2013). Additionally, SN1 and LP1 were found to be negatively correlated with the ability of ramie to thrive under poor soil conditions. This may be the strategy by which ramie copes with the mid-season harvest. A lower SN1 and LP1 could reduce the amount of nutrients removed by the mid-season harvest. Overall, N translocation is more important than P translocation, followed by K translocation. This is consistent with a previous work showing that ramie growth is sensitive to N and P deficiency but not to K deficiency (Liu et al., 2012).

### Effect of Soil Microbial Structure on the Ability of Ramie to Thrive Under Nutrient-Deficient Conditions

An additional aim of this study was to clarify the role of the soil microbial community on the ability of ramie to tolerate poor soil. The enrichment of the beneficial bacterial communities aided in the adaptation of ramie to poor soil conditions. Of the six significantly enriched bacterial genera, most have been shown to facilitate plant growth. For example, *Bradyrhizobium* are N-fixing bacteria that can minimize the effects of nitrogen deficiency on plant growth (Hara et al., 2019; Ormeño-Orrillo and Martínez-Romero, 2019). *Gaiella* and *norank_o_Gaiellales* are both *Actinobacteria*, which can promote plant growth by increasing nutrient availability and assimilation (Franco-Correa and Chavarro-Anzola, 2016; Novello et al., 2017; Severino et al., 2019). *Candidatus_Solbacter* can reduce moisture and nutrient fluctuations in stressful soil environments through the production of biofilms (Pearce et al., 2012). It can thus improve the ability of plants to withstand extreme environmental variation in moisture and nutrient availability (Challacombe et al., 2011). Although the exact functions of *norank_f_SC-I-84* and *nornak_o_IMCC26256* remain unclear, this study confirms that they are closely tied to ramie growth under nutrient-deficient conditions. In contrast to the positive effect of bacterial communities, a reduction in harmful fungal communities is another strategy by which ramie can adapt to poor soil conditions. Of the significantly related fungal genera, *Cladosporium* can produce antimicrobial metabolites (Yehia et al., 2020) that can kill certain types of beneficial bacteria such as N-fixing bacteria. *Aspergillus* can induce plant diseases that can reduce plant yields (Larbi-Koranteng et al., 2020). *Cladorrhinum* negatively affected ramie growth in this study, in contrast to its potential utility for biocontrol and its ability to promote plant growth (Carmarán et al., 2015). This may stem from the negative effect of some undetected species, but this hypothesis requires confirmation in future trials.

## Conclusion

Based on two long-term (8–9 years) field trials of ramie cultivation, the genotypes of Duobeiti 1 and Xiangzhu XB were shown to be highly tolerant of poor soil. Low nutrient content in the stem and leaves and N and P retranslocation to the root at the end of the growing season are strategies underlying the ability of these genotypes to adapt to poor soil conditions. The enrichment of beneficial bacterial communities and the decrease in harmful fungal communities also help ramie plants adapt to poor soil conditions.

## Data Availability Statement

The datasets presented in this study can be found in online repositories. The names of the repository/repositories and accession number(s) can be found below: CNCB-NGDC (accession: CRA003958; https://bigd.big.ac.cn/gsa).

## Author Contributions

YJ and HX designed the experiments. SW performed the experiments and wrote the manuscript. SW and SX analyzed the data. YJ, SX, and YI reviewed and edited the manuscript. All authors read and approved the manuscript for submission.

## Conflict of Interest

The authors declare that the research was conducted in the absence of any commercial or financial relationships that could be construed as a potential conflict of interest.

## Footnotes

1 http://faostat.fao.org

2 www.majorbio.com
